# Wild gelada monkeys detect emotional and prosocial cues in vocal exchanges during aggression

**DOI:** 10.1371/journal.pone.0323295

**Published:** 2025-05-14

**Authors:** Luca Pedruzzi, Martina Francesconi, Alice Galotti, Bezawork Afework Bogale, Elisabetta Palagi, Alban Lemasson

**Affiliations:** 1 EthoS (Ethologie Animale et Humaine) - U.M.R, Université de Rennes, Université de Normandie, CNRS, Rennes, France; 2 Unit of Ethology, Department of Biology, University of Pisa, Pisa, Italy; 3 Department of Zoological Sciences, College of Natural and Computational Sciences, Addis Ababa University, Addis Ababa, Ethiopia; 4 Natural History Museum, University of Pisa, Calci, Pisa, Italy; 5 Institut Universitaire de France, Paris Cedex, France; Ariel University, UNITED KINGDOM OF GREAT BRITAIN AND NORTHERN IRELAND

## Abstract

Recognizing vocal behaviours intended to benefit others is a crucial yet understudied social skill. Primates with rich vocal repertoires and complex societies are excellent models to track the evolution of such capacity. Here, we exposed wild geladas (*Theropithecus gelada*) to vocal exchanges between unfamiliar female victim screams and male affiliative calls. The stimuli were arranged in sequences either simulating vocal affiliation towards victims (scream-affiliative call) or violating such order (affiliative call-scream), with varying emotional arousal conveyed by the affiliative call type. Measuring gazing activity towards the loudspeaker and the interruptions of feeding, we show that monkeys were sensitive to the sequential order in vocal exchanges as well as to the emotional arousal conveyed by affiliative calls. Our field study suggests a prosocial use of vocalizations in wild monkeys and reveals that foundational cognitive elements for processing vocal exchanges as meaningful third-party interactions may have existed in our common ancestors with monkeys.

## Introduction

Across human and non-human social animals, vocalizations play a crucial role in facilitating appropriate responses to others’ needs [1–5]. In particular, they do so by conveying a wide range of affective states through both within-call type variability, such as changes in pitch, duration, or amplitude, and between-call type variability, where different call categories signal distinct emotional or social contexts [1–5]. Vocal signals serve as vital cues for coordinating group activities, reinforcing social bonds, and conveying intentions to help or cooperate, ultimately fostering group cohesion [6]. Moreover, attending to a vocal exchange between two subjects can inform about their relationship and the nature of the interaction (e.g., indirect social information processing), especially in complex social systems where visual contact can be constrained. By simply listening to call exchanges between two individuals, several primates can indeed make inferences and obtain salient social information about subject identity and directedness of the interaction [7], rank and kinship of the interacting agents [8–10], and context of call production [11]. Playback experiments show that some animal species demonstrate a certain degree of awareness of a third-party conflict even when solely exposed to vocal cues [3,8,12,13] thus making aggression and post-conflict behaviour excellent models to study vocal complexity and prosocial tendencies [2,14].

In some cases, vocalizations can also actively elicit or function as prosocial behaviours, as certain vocal signals may act as affiliative acts that promote social bonding or solicit cooperative actions from others [14–20]. Prosociality is a fundamental building block of human and non-human tolerant societies [21] and comprises all those intentional behaviours expressed to benefit and help others [22]. The immediate factors influencing prosocial acts are multifaceted, encompassing direct requests from the beneficiary, potential benefits for the donor, and robust social bonds between the interacting agents [23]. More in general, social (e.g., cooperative alloparental breeding) and ecological (e.g., resource distribution) factors that characterize different species play a key role in the evolution of prosocial and cooperative behaviours [24]. While experimental studies suggest that some non-human animals possess cognitive abilities to recognize prosocial from non-prosocial groupmates based on past experience and social cues (primates^,^ [25], corvids, [26]), scarce data are present on animals’ ability to distinguish prosocial behaviours solely based on vocal cues. For instance, dwarf mongooses (*Helogale parvula*) and marmosets (*Callithrix jacchus*) have been shown to obtain information regarding the cooperativeness of a subject from its vocalisations [27,28]. However, the two cases might be explained by the cooperative breeding system of the two species, where individuals rely on cooperative acts from group members; this could enhance the sensitivity to social cues and might have selected fine strategies to punish or reward groupmates according to their cooperation propensity [27,28].

In many primates, victims of aggression often emit screams, stereotyped noisy drawn-out calls signalling the subject distress which can sometimes alert or recruit prosocial responses and facilitate reconciliation and consolation [12,15–17,20,29]. Vocalisations produced by bystanders of aggression can sometimes act as a form of affiliative and/or prosocial act towards victims, such as calming or comforting vocalizations that may facilitate social bonding [14,19,30,31]. Positive vocalizations, such as coos, grunts, or other affiliative calls [17,19], can indeed trigger neuroendocrine responses that promote social cohesion and reduce stress, mimicking the calming effects of physical touch (humans, [32], rats, [33]). These vocal behaviours reflect the complexity of primate communication, where vocalizations play an active role in regulating social dynamics and facilitating cooperation [6].

Here, we use a playback paradigm with simulated vocal exchanges during aggression in a wild population of geladas (*Theropithecus gelada*) to investigate their ability to extract social information from a third-party vocal interaction. Geladas, a monkey species endemic to Ethiopia, live in complex multi-level societies (basic group units: One-Male Unit, OMU) with high levels of intra-unit cohesion and inter-unit tolerance [34–36]. Conflict-resolution strategies adopted by the species involve both reconciliation and spontaneous triadic affiliation towards victims [37,38]. Geladas show a richer vocal repertoire compared to other phylogenetically close cercopithecines [29,39–42] and their socio-communicative complexity has often been considered a precursor for that of humans [43,44]. For these reasons, among primates, geladas are optimal candidates to study the recognition of compositional order and affective states in third-party vocal exchanges. Concerning affiliative calls, not only do geladas produce grunts, low-intensity affiliative contact calls [45] commonly shared with *Papio* baboons, but they have also evolved unique derived positive calls mostly produced by males such as moans and wobbles [29,41]. Moans are derived long drawn-out affiliative grunts (elongated version of a grunt) produced by both inhalation and exhalation [46]. Moans have been described as more salient and attractive than grunts for females [29,46], possibly conveying higher emotional arousal [1,2]. The need for cross-sex bonding in their unique social system has possibly driven gelada male vocal complexity [34], with evidence of control in the rhythm and melody during vocal exchanges with females in emotionally aroused contexts [47]. Vocal sequences including moans are often produced by leader males to their OMU’s females in contexts in which the need to maintain proximity is highest (e.g., moving, presence of other group units) and often lead to grooming [34]. Data on the use of vocal signals during aggression are relatively limited, and, more broadly, there is ongoing debate about whether vocalizations in non-human primates primarily serve communicative functions or are largely involuntary expressions of emotions [48,49]. In geladas, although intersexual communication has been studied in affiliative [34] and mating [40] contexts, research is lacking on male-female vocal exchanges during negative contexts. Indeed, few studies only suggest that moans and grunts can be used after aggression to affiliate with victims, either by the aggressors or by uninvolved third-party individuals [29,37,38], highlighting a gap in the literature.

**Fig 1:**
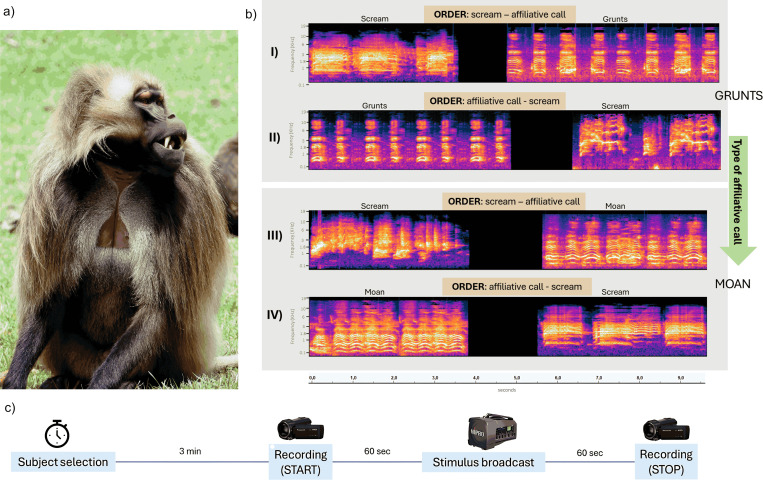
A study animal and examples of vocal exchanges used as stimuli. a) Picture of an adult gelada male in the study area (picture by EP); b) graphical representations of the four conditions of simulated vocal exchanges used as stimuli during the experiments are represented, with four examples of spectrograms (obtained on Audacity© v. 3.3.2); c) Schematic representation of the different steps of the experimental procedure. See also S1 Fig.

In the present study we use a field playback experiment with vocal exchanges between screams emitted by victims of aggression and different affiliative vocal contacts produced by males (e.g., the aggressor or a third-party subject). In particular, we employ a paradigm analogous to the violation of expectation, in which subjects are presented with stimuli that defy their expectations, eliciting varying signs of surprise, vigilance, or attention, generally measured using the gazing behaviour or behavioural interruptions as markers [50–52] (see section Video coding and statistical analyses). These expectations are considered indicative of the mental principles the subjects hold about their surrounding environment [53]. The method has been validated as an effective tool for uncovering the functional role and underlying rules governing primate vocal exchanges [50,51]. Specifically, we exposed the animals to acoustic sequences either simulating vocal affiliation towards victims (when a victim’s scream was followed by an affiliative vocal contact, scream-affiliative call) or violating such order (affiliative call-scream). Depending on the type of affiliative call used by the male, we either have a low (grunts) or high (moans) emotional arousal conveyed by the call (Fig 1). We broadcast calls from unfamiliar individuals to prevent any bias deriving from previous experience with socially bonded callers [46]. This allows evaluating monkey ability to generally recognize the intrinsic nature of the message conveyed by the signal. We posited that if geladas recognize the sequential order of a vocal exchange between the scream of a female victim and the affiliative vocal contact of a male (*Hypothesis A*), we expect vocal exchanges violating a socially positive order (i.e., when a positive affective call is followed by a negative one) to attract more interest (in terms of gazing) compared to vocal exchanges simulating vocal affiliation towards the victim (i.e., solved aggression) (*Prediction A*). We expect monkeys to show some sort of sensitivity (shown by different levels of interest) also to the prosocial effort expressed by the emotional arousal of the positive vocalisation emitted [2] (*Hypothesis B*). In this view, we predict vocal exchanges containing male moans (i.e., moan-scream/scream-moan sequences), to elicit prolonged responses than those containing male grunts (i.e., grunts-scream/scream-grunts sequences), affiliative calls expressing lower arousal (*Prediction B*).

## Materials and methods

### Experimental model and subject details

The playback experiments were conducted between February and April 2024 in the unprotected area of Debre Libanos, in the central highlands of Ethiopia (Oromia Region, Northwest Shewa zone) [54,55]. Specifically, 11 One-Male Units (OMUs) were followed from 8:00 AM to 16:00 PM during the study period (for a total of 391 hours), as they daily visited a large grazing area in Set Deber, one of the sites in Debre Libanos inhabited by gelada groups [55]. The study area (S1 Fig) allowed for optimal conditions for playback experiments (e.g., comparable microhabitat conditions across experiments). We could test 10 fully-grown adult male subjects which were the alpha males of 10 of the 11 OMUs followed; the subjects were individually recognized and familiar to human presence, thus allowing researchers to be in close proximity. The selection of adult male subjects was primarily driven by practical fieldwork considerations, including their easier and unequivocal identification, the feasibility of consistent tracking, and their ability to reliably adhere to the experimental paradigm and necessary procedural precautions (see Experimental Procedure). Moreover, adult males are the primary producers of derived affiliative calls [34], playing a key role in regulating group dynamics. As a result, they are theoretically the most attentive to variations in vocal sequence structure, making them particularly relevant for investigating sensitivity to syntactic changes in our pilot playback experiment.

### Ethical statement

Formal approval for the playback experiments was sought and obtained from the Bioethical Committee of the University of Pisa (OPBA, n. 14/2023). Additionally, research procedures strictly adhered to the laws and approved guidelines set forth by the Ethiopian Wildlife Conservation Authority (EWCA).

### Stimuli preparation

To prepare the playback stimuli, LP and MF collected vocalisations produced by geladas living in the captive colony at NaturZoo Rheine (Germany) in April-May 2023 [42]. Vocalisations were collected with a directional microphone (Sennheiser© MKE600) connected to a handy recorder (ZOOM H5©, sample rate: 44,100 Hz, resolution: 16-bit, wav format) during spontaneous interactions (distance from the animals varied from 5 to 15 m). All subjects producing the stimuli (5 adult leader males, 40 females from the different OMUs; subjects individually recognized by LP and MF) were thus totally unfamiliar to the study subjects. We selected three different call types among the gelada vocal repertoire: male grunts, male exhale moans, and female screams [29]. These vocalisations can occur during aggression; from the observational data we collected at NaturZoo Rheine, we recorded 103 agonistic interactions during which both moans/grunts and scream were produced; in 58 cases the scream of a victim was followed by the affiliative call of a male, whereas in 45 events the affiliative call of the male preceded the scream of the female. During the recording phase, we also measured the loudness (decibel, dB) of the three different vocalisations (mean ± SD = 59.1 ± 8.6 dB) with a professional sound meter (SLM-25, Gain Express Holdings©). Only stimuli with a high sound-to-noise ratio were kept (e.g., no birds or overlap with vocalisations from other geladas).

The recordings were then edited using Audacity© software (version 3.3.2) to create a pool of grunts (always produced in series, on average four in the present case), moans, and screams used to then build the final stimuli. All stimuli contained one scream each but differed in the affiliative call (and relative order) in the sequence. To prevent any bias in loudness variations, we normalized the amplitude of all calls and, before the beginning of the experiments and not in the experimental area, we checked the speaker volume to reach about 60.0 Db [51,56] at the tested subject location (15–20 metres, see Experimental procedure). To homogenize the amount of acoustic information conveyed by the two positive call types, the duration of grunt series was adjusted to that of moans by repeating each grunt sequence twice (totalizing 8 grunts *vs* 1 continuous moan). The pool of calls used to build stimuli thus comprised a total of 40 screams (duration: mean ± SE = 5.214 ± 0.236 seconds), 20 grunt series (each composed of 8 exhale and inhaled grunts, duration: mean ± SE = 5.579 ± 0.248 seconds), and 20 exhale moans (duration: mean ± SE = 5.372 ± 0.194 seconds). The five males equally contributed to the calls recorded (grunt series and moans), preventing pseudo-replication. The calls were randomly selected to build four different playback stimuli then randomly assigned to the 10 study subjects (for a total of 40 stimuli broadcast in 40 experimental sessions). The four stimuli to which each tested animal was exposed to contained four affiliative call series produce by four captive males (identity randomly assigned) and four screams produced by four captive females (identity randomly assigned) (Fig 1). A given vocalisation was thus never used twice during the entire experimental period. The four stimuli were i) scream – grunt series (*Sequential order*; *Type of affiliative call*: Grunts), ii) grunt series – scream (*Sequential order*; *Type of affiliative call*: Grunts) iii) scream – moan (*Sequential order*; *Type of affiliative call*: Moan), iv) moan – scream (*Sequential order*; *Type of affiliative call*: Moan) (Fig 1). The silence duration from the first to the second vocalisation comprised in each stimulus randomly ranged from 1.5–2.5 seconds, believed to be a realistic latency time with which a male would react to a female scream. The mean duration of the total stimuli used was 10.2 seconds (SE: 0.6 s). For a given subject, stimuli duration did not differ either between the two conditions of *Type of affiliative call* (grunts *vs* moan, Mann-Whitney-Wilcoxon Test, n_1_ = n_2_ = 20, W = 197, p = 0.95) or between the two conditions of *Sequential order* (scream-affiliative call *vs* affiliative call-scream, Mann-Whitney-Wilcoxon Test, n_1_ = n_2_ = 20, W = 213, p = 0.73). We checked for possible intra-call variability between experimental conditions. For a given subject, no difference in the fundamental frequency (f0), generally indicating emotional arousal [1,4,57], was detected in the screams used in the two conditions of *Type of affiliative call* (grunts *vs* moan, Mann-Whitney-Wilcoxon Test, n_1_ = n_2_ = 20, W = 208, p = 0.84) and *Sequential order* (scream-affiliative call *vs* affiliative call-scream, Mann-Whitney-Wilcoxon Test, n_1_ = n_2_ = 20, W = 188.5, p = 0.77). Similarly, the f0 of grunt series used in the two conditions of *Sequential order* did not differ (scream-affiliative call *vs* affiliative call-scream, Mann-Whitney-Wilcoxon Test, n_1_ = n_2_ = 10, W = 70, p = 0.14), as well as that of moans in the two conditions of *Sequential order* (scream-affiliative call *vs* affiliative call-scream, Mann-Whitney-Wilcoxon Test, n_1_ = n_2_ = 10, W = 34, p = 0.25) [56].

### Experimental procedure

Several precautions were taken to limit habituation and confounding factors [16,46,58]. The environmental conditions and study subjects’ habituation to the researchers allowed the distance gelada-speaker to be kept as constant as possible during the different playback sessions (~15 metres). The speaker (MiPRO© MA-100 single channel Personal Wireless PA system) was positioned so that the sound would come from the direction where no other groups were present not to simulate the vocal presence of an unfamiliar male in a known group. No aggression took place in the 30 minutes before and no affiliative calls were audible by researchers in the 10 minutes before each playback. During experiments, one observer (LP) played the stimulus (in.wav format) at distance via Bluetooth connected to the speaker hidden in vegetation (S1b Fig). Another experimenter (MF, AG), generally visible to the tested gelada, video-recorded the animal (SONY© handy-cam Full HD FDR-AX43A) in the 60 seconds before and after the sequence broadcast. After the experimenter and the loudspeaker were set, we waited at least three minutes before starting the trial. This latter experimenter’s direction was of least 90° shifted compared to the direction of the acoustic stimulus. Each subject experienced the four conditions in a random order (half sessions in the morning, 9–12am, half in the afternoon, 1–4pm). Subjects were always tested during feeding, as the experimental area was a grazing area (S1 Fig) visited by the study groups mostly for feeding, when not involved in social interactions, and with microhabitat visibility qualitatively similar across sessions. We recorded whether other OMUs were in proximity (within ~ 50 metres) to the tested subject. The subject had to remain visible and no event possibly affecting its vigilance (e.g., arrival of new group, aggression, vocalisations by other group members) had to occur during the whole recording. No more than two playbacks were conducted per day and a condition was never played more than once per day. When a playback occurred, the identity of non-tested males who could potentially hear the stimulus was coded so that we were sure that a minimum of 48 hours separated instances in which a given gelada male could hear two stimuli. Moreover, to familiarize the animals with equipment and the presence of researchers, situations comparable to “mock” experiments were conducted in which observers and equipment were positioned as in actual experiments, but no stimulus was broadcast (the study groups were daily followed and recorded during the whole experimental period).

### Video coding and statistical analyses

Video recordings were analysed frame-by-frame (PotPlayer©, accuracy 0.02 sec), coding the following behaviours: i) looking at the direction of the speaker, ii) self-directed behaviours (scratching, self-grooming, proxy for anxiety state in primates [59]) (see S1 Table for definitions and operationalization). Every instance in which the tested subjects turned their head towards the direction of the speaker was coded as looking at. Measures of the time subjects change head orientation after playback are commonly used to evaluate their general interest in a given stimulus in non-human primates [16,60]. Moreover, since geladas evolved a highly specialized graminivorous diet [61], their feeding implies a seated position and the deployment of a series specialized hand movements [62] with their head facing the ground [63], this allowed us to code for the iii) time in which the subject interrupted feeding (e.g., interrupting hand movements and straightening up the head, only instances of feeding interruption for > 0.5 seconds were considered reliable stops of the activity) as a possible proxy for vigilance state of the subject. Indeed, while vigilance seems relatively cost free for upright feeders eating food that requires little manipulation [52], this is not the case for the gelada unique feeding strategy [61–63]. To control for individual variations in vigilance or attentional state, behaviours were coded in the 60 seconds before stimulus onset and in the 60 seconds after the whole stimulus broadcasting, thus after the animal could hear the entire sequence [46]. Behaviours were coded also before stimulus onset to measure the increase/decrease of interest towards the speaker area after the stimulus compared to before [16,50]. No vocalisations by the study subjects were recorded during the experimental sessions, neither before nor after stimulus presentation. To evaluate the first interest towards the stimulus, we calculated the latency to return to the head position before the stimulus (as in Pougnault et al. [50]), as subject started looking at the speaker before the end of the whole acoustic sequence. Then, to evaluate the prolonged response elicited by the stimulus, we measured the total time spent gazing and calculated the mean duration per gaze, as well as measured the time spent in self-directed behaviours, and in which they interrupted feeding (all measured in 60 seconds after – 60 seconds before stimulus broadcasting). The coder (LP) was blind (muted videos labelled by MF) to the condition of the videos. Inter-observer reliability was assessed with a second coder (AG) who coded 25% of videos blind to the conditions of the playback sessions and who was in significant agreement with the first coder (for the four variables, Intraclass Correlation Coefficient (ICC) ≥ 0.83, p < 0.001[64])

*Model 1–5 –* We ran five GLMMs with *Duration of first interest to the loudspeaker* (*Model 1*), *Total gaze duration towards the loudspeaker* (*Model 2*), *Mean duration per gaze to the loudspeaker* (*Model 3*)*, Duration of stop in feeding activity* (*Model 4*), and *Self-directed behaviours* (*Model 5*) as response variables using a Gaussian distribution, log-transforming the response variables for *Model 1*–*3* after checking for model fit and diagnostics (DHARMa [65]). The response variables are measured in tenths of seconds. Apart from the response variable, *Model 1*–*5* were equally built: the *subject ID* was included as random factor, whereas the fixed factors considered were the *Sequential order* (scream-affiliative call/affiliative call-scream), the *Type of affiliative call* (grunts/moan) in the vocal exchange, the presence of *Other OMUs in proximity*, and the *Trial number*.

All GLMMs allowed possible zero-inflation issues (zero values were present in all response variables) thanks to the use of the glmmTMB package [66]; however, we did not model the zero-inflated part of the data including further model components not to overcome optimal observations-predictors ratio in the models [67]. We checked for multicollinearity [67] in the GLMMs using the ‘check_collinearity’ function (R package performance 0.4.4). ‘Low correlation’ was found for all the fixed factors in the four models (VIF range: 1.06–1.65). We tested the models’ significance by comparing the full with the control model (i.e., only including random and control factor(s)) through the Likelihood Ratio Test (LRT, Anova with the ‘Chisq’ argument) and we then estimated the p-values of each predictor running LRTs between the full model and the model not containing that predictor [68]. To check the models fit and possible overdispersion issues the package DHARMa 0.3.3.0 [65] was used. The GLMMs were not over-dispersed (dispersion range: 1.02–1.07, p-value range: 0.66–0.87), no outliers were detected (p-value range = 0.27–1), and normality of the residuals was confirmed via visual inspection of Q-Q plots (Kolmogorov-Smirnov test, p-value range: 0.23–0.73). All the analyses were carried out using R_studio_ (http://www.r-project.org).

## Results

A total of 40 experimental sessions were conducted on 10 adult gelada males, with each individual experiencing the four different conditions, i) scream – grunt series, ii) grunt series – scream iii) scream – moan, iv) moan – scream; (Fig 1).

**Fig 2:**
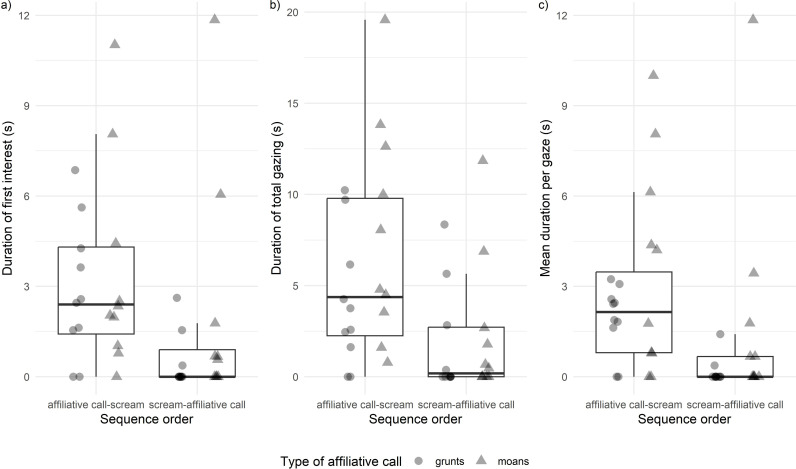
The effect of stimulus sequential order on gelada gazing behaviour. Influence of the *Sequential order* of the simulated vocal exchange on a) *Duration of first interest towards the loudspeaker* (measured in seconds) (*Model 1*: χ^2^ = 11.646, *P* < 0.001)*,* b) *Duration of total gazing towards the loudspeaker* (measured in seconds) (*Model 2*: χ^2^ = 17.36, *P* < 0.001), and c) *Mean duration per gaze towards the loudspeaker* (measured in seconds) (*Model 3*: χ^2^ = 14.718, *P* < 0.001). Subjects’ ID are represented with different colours and the *Type of affiliative call* of the stimulus is indicated by the point shape (triangle = grunts; circle = moans). The boxes display the median value and first and third quartiles, whiskers are extended to the most extreme value inside the 1.5-fold interquartile range.

*Model 1 – Duration of first interest towards the loudspeaker* The full model was significantly different from the control one (χ^2^_4_ = 14.79, *P* = 0.005). The *Sequential order* and *Type of affiliative call* of the stimulus significantly affected the *Duration of first interest towards the loudspeaker*, as the latency with which geladas turned their head back to the original position after stimulus broadcasting was shorter when the stimulus had a sequential order simulating vocal affiliation to the victim (*Sequential order*: | Coefficient|= 1.821, χ^2^ = 9.91, *P* = 0.001, Table 1, Fig 2a) and when the affiliative call in the stimulus had higher emotional arousal (*Type of affiliative call*: | Coefficient|= 1.604, χ^2^ = 7.828, *P* = 0.005, Table 1).

**Table 1:** Duration of first interest to the loudspeaker.

Fixed Effects	Coeff	SE	χ^2^	df	*P*
***Model 1 - *** *Duration of first interest to the loudspeaker*					
Intercept	2.540	0.948	–	–	–
**Tested variables**					
Type of affiliative call (moans)	1.604	0.948	7.828	1	**0.005**
Sequential order (scream-affiliative call)	-1.821	0.579	9.908	1	**0.001**
Trial number	-0.304	0.254	1.434	1	0.231
Other OMUs in proximity (Yes)	0.671	0.660	1.034	1	0.309

Random factors: subject ID, Variance=1.357, SD=1.165.

Estimated parameters (Coeff), Standard Error (SE), and results of the Likelihood Ratio Tests (χ^2^) of Model 1. Significant *P* values are bold; df = degree(s) of freedom; - = not applicable. Estimate ± SE refers to the difference of the response between the reported level of this categorical predictor and the reference category of the same predictor. n_playbacks _= 40 and n_subjects _= 10.

**Fig 3:**
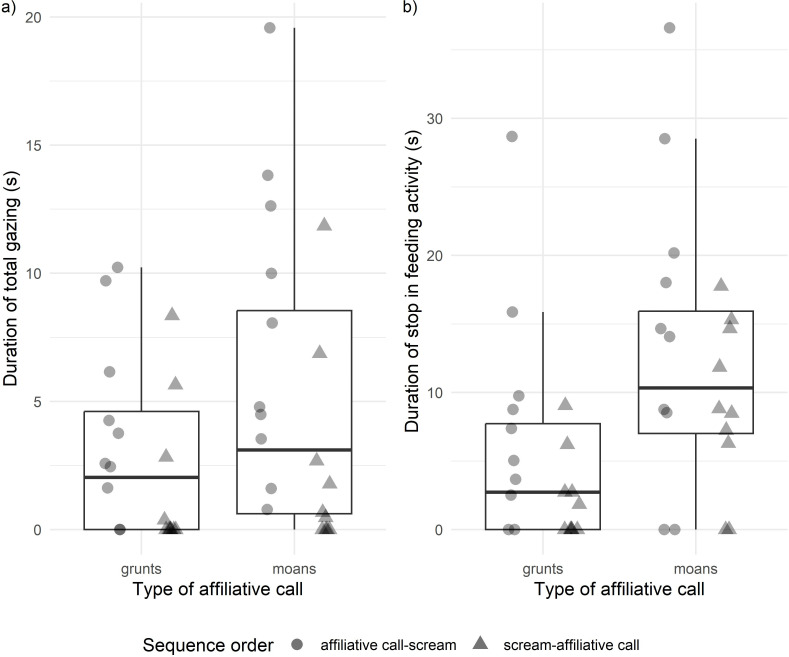
The effect of the emotional arousal of the affiliative call in the sequence on gelada gazing behaviour and feeding interruption. Influence of the *Type of affiliative call* of the stimulus (grunts *vs* moans) on a) *Duration of total gazing towards the loudspeaker* (seconds) (*Model 2*: χ^2^ = 3.994, *P* = 0.046), b) *Duration of stop in feeding activity* (seconds) (*Model 5*: χ^2^ = 5.65, *P* = 0.017). Subjects’ ID are represented with different colours and the *Sequential order* of the vocal exchange is indicated by the point shape (triangle = scream-affiliative call; circle = affiliative call-scream). The boxes display the median value and first and third quartiles, whiskers are extended to the most extreme value inside the 1.5-fold interquartile range.

*Model 2 – Duration of total gazing towards the loudspeaker* (measured during 60 seconds after – during 60 seconds before stimulus presentation, see Methods). The full model was significantly different from the null model (χ^2^_4_ = 20.13, *P* < 0.001). The *Sequential order* and the *Type of affiliative call* of the stimulus significantly affected the *Duration of total gazing towards the loudspeaker*. Geladas looked for longer primarily when stimuli had the affiliative call-scream sequential order (*Sequential order*: | Coefficient|= 2.695, χ^2^ = 18.10, *P* < 0.001, Table 2, Fig 2b) and secondarily when stimuli contained a positive call expressing high emotional arousal (*Type of affiliative call*: | Coefficient|= 1.274, χ^2^ = 4.122, *P* = 0.042, Table 2, Fig 3a).

**Table 2:** Duration of total gazing towards the loudspeaker.

Fixed Effects	Coeff	SE	χ^2^	df	*P*
***Model 2 - *** *Duration of total gazing towards the loudspeaker (60s after - 60s before stimulus)*					
Intercept	3.939	1.048	–	–	–
**Tested variables**					
Type of affiliative call (moans)	1.274	0.627	4.122	1 1	**0.042**
Sequential order (scream-affiliative call)	-2.695	0.633	18.098	1	**<0.001**
Trial number	-0.529	0.279	3.604	1	0.058
Other OMUs in proximity (Yes)	-0.141	0.720	0.038	1	0.844

Random factors: subject ID, Variance=1.774, SD=1.332.

Estimated parameters (Coeff), Standard Error (SE), and results of the Likelihood Ratio Tests (**χ**^**2**^) of Model 2. Significant *P* values are bold; df = degree(s) of freedom; - = not applicable. Estimate ± SE refers to the difference of the response between the reported level of this categorical predictor and the reference category of the same predictor. n_playbacks _= 40 and n_subjects _= 10.

*Model 3 – Mean duration per gaze towards the loudspeaker* (measured during 60 seconds after – during 60 seconds before stimulus presentation, see Methods)*.* The full model was significantly different from the null model (χ^2^_4_ = 17.42, *P* = 0.001). The *Sequential order* of the stimulus significantly affected the *Mean duration per gaze towards the loudspeaker*, as geladas looked for longer towards the speaker when the stimulus violated the sequential order simulating vocal affiliation to the victim (*Sequential order*: | Coefficient|= 1.778, χ^2^ = 19.569, *P* < 0.001, Table 3, Fig 2c). On the other hand, the *Type of affiliative call* in the stimulus did not affect the *Mean duration per gaze* (*Type of affiliative call*: χ^2^ = 3.820, *P* = 0.053, Table 3).

**Table 3:** Mean duration per gaze towards the loudspeaker.

**Fixed Effects**	**Coeff**	**SE**	**χ** ^**2**^	**df**	***P***
***Model 3 -*** *Mean duration per gaze towards the loudspeaker (60s after - 60s before stimulus)*					
Intercept	2.158	0.646	–	–	–
**Tested variables**					
Type of affiliative call (moans)	0.778	0.398	3.820	1	0.051
Sequential order (scream-affiliative call)	-1.778	0.402	19.569	1	**<0.001**
Trial number	-0.023	0.178	0.017	1	0.897
Other OMUs in proximity (Yes)	0.463	0.452	1.047	1	0.306

Estimated parameters (Coeff), Standard Error (SE), and results of the Likelihood Ratio Tests (**χ**^**2**^) of Model 3. Significant *P* values are bold; df = degree(s) of freedom; - = not applicable. Estimate ± SE refers to the difference of the response between the reported level of this categorical predictor and the reference category of the same predictor. n_playbacks _= 40 and n_subjects _= 10.

*Model 4 – Duration of stop in feeding activity* (measured during 60 seconds after – during 60 seconds before stimulus presentation, see Methods)*.* The full model significantly differed from the null one (χ^2^_4_ = 13.62, *P* < 0.001). Geladas stopped their feeding activity for longer when the affiliative call in the vocal exchange was a moan (*Type of affiliative call*: | Coefficient|= 65.60, χ^2^ = 7.42, *P* = 0.006, Table 4, Fig 3b) and when the *Sequential order* of the stimulus did not simulate vocal affiliation towards the victim (*Sequential order*: χ^2^ = 4.79, *P* = 0.029, Table 4).

**Table 4:** Duration of stop in feeding activity.

**Fixed Effects**	**Coeff**	**SE**	**χ** ^**2**^	**df**	***P***
***Model 5 -*** *Duration of stop in feeding activity (60s after - 60s before stimulus)*					
Intercept	119.38	35.86	**–**	**–**	**–**
**Tested variables**					
Type of affiliative call (moans)	65.60	24.08	7.421	1	**0.006**
Sequential order (scream-affiliative call)	-53.13	24.28	4.789	1	**0.029**
Trial number	-12.58	10.60	1.409	1	0.235
Other OMUs in proximity (Yes)	-17.54	25.05	0.490	1	0.484

Random factors: subject ID, Variance=1.11*10^-31^, SD=3.25*10^-16^.

Estimated parameters (Coeff), Standard Error (SE), and results of the Likelihood Ratio Tests (χ2) of Model 5. Significant P values are bold; df = degree(s) of freedom; - = not applicable. Estimate ± SE refers to the difference of the response between the reported level of this categorical predictor and the reference category of the same predictor. n_playbacks _= 40 and n_subjects_ = 10.

*Model 5 – Self-directed behaviours (60s after - 60s before stimulus)* The full model did not significantly differ from the control one (χ^2^_4_ = 5.38, *P* = 0.25). None of the fixed factors included thus affected the variability of the time study subjects spent in self-directed behaviours.

## Discussion

**Fig 4:**
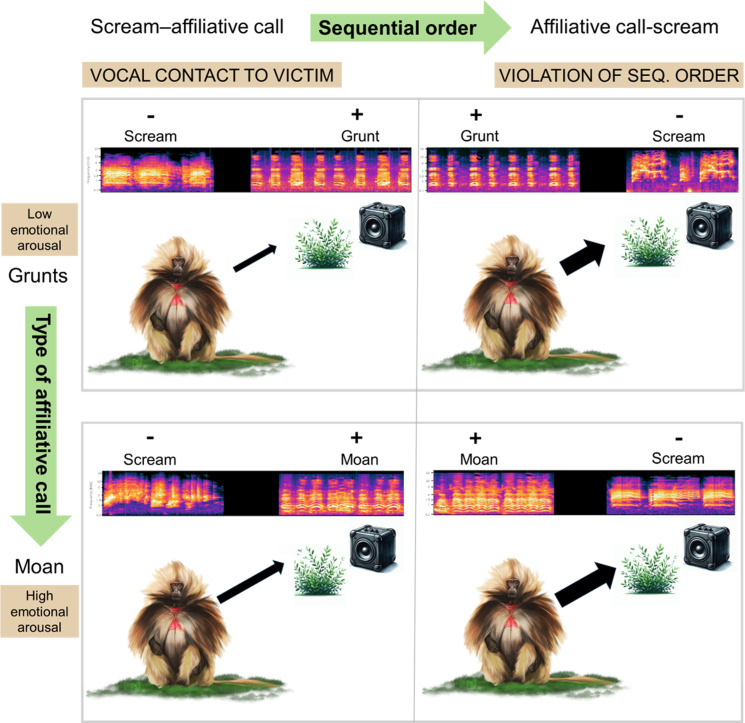
Graphical abstract of the playback experiment. Geladas showed more interest towards stimuli violating a positive sequential order as well as after stimuli with calls of high emotional arousal. Gelada monkeys seem to show cognitive ability to recognize vocal affiliation directed at victims.

To comprehend the evolutionary origins of the ability to extract salient social information from third-party vocal interactions, it is essential to gather comparative data from species that exhibit social multilevel complexity and vocal richness. Geladas, with their intricate social structures and vocalization diversity, provide an ideal model for such studies. Here, we exposed wild geladas to vocal interactions between unfamiliar female victim screams and male affiliative calls with sequential order either simulating vocal affiliation towards the victim (scream-affiliative call) or violating such order (affiliative call-scream), using as affiliative call either grunt series (low emotional arousal) or moans (high emotional arousal) (Fig 1). Geladas looked for longer towards the loudspeaker when the vocal exchange violated a possible conflict resolution (*Prediction A* supported). Moreover, they also seemed sensitive to the affiliative call used towards victims as study subjects interrupted feeding activity for longer and looked for longer towards the loudspeaker in response to vocal exchanges containing affiliative moans compared to grunt series (*Prediction B* supported) (Fig 4).

A growing body of literature is uncovering how some primate species can compositionally combine calls to create new meanings and vocal complexity [69–73]. Variability in communication can also be obtained by changing the order of calls composing vocal sequences [69,74] as listeners can then attend to the referential changes induced by such permutations [75]. The capacity of extracting meaning from the entire vocal sequences is crucial for survival in social groups [71]. This has been so far studied in the vocal sequences produced by one subject, but such processing capacity might extend to listeners attending to third-party vocal exchanges between two subjects. It is indeed known that primates respond differently to third-party vocal exchanges respecting *vs* not respecting different social rules [50,51,76]. Here, our results may support this idea, suggesting that geladas possess sophisticated communicative abilities when processing third-party vocal interactions.

Here, as the male vocally contacting the female could have been interpreted by the listeners as either the aggressor or as a bystander, our results can be interpreted in two main ways. On the one hand, animals listening to the playbacks might have interpreted them as reconciled *vs* not reconciled aggression and might have been sensitive to the resolution of the conflict. On the other hand, they could have interpreted them as if a bystander was consoling a victim, demonstrating to be aware of a prosocial role of affiliative vocalizations. In any case, consistent with the species’ vocal and social complexity, our findings suggest that geladas can extract meaningful social information (i.e., emotional arousal conveyed to the victim of an aggression, presence of vocal affiliation and sequential outcome of a conflict) from acoustic cues and might possibly distinguish between prosocial and non-prosocial vocal exchanges. Importantly, our observational data on the natural occurrence of the vocal sequences used in our experiment (see Methods) indicate that both scream-affiliative call and affiliative call-scream can occur during aggression in geladas; this suggests that the higher interest paid to stimuli violating vocal affiliation towards a victim was not due to such order merely being unusual or absurd, but rather to the sensitivity to the outcome of a conflict in a potentially nearby group unit.

Our data can add important insights into the gelada complex social and vocal dynamics and, more in general, to social eavesdropping of animals indirectly obtaining information about intra- and intergroup conflicts [13]. In species living in complex social systems, such as geladas, acoustic cues can be especially valuable sources of social information. These signals, produced during within- or between-group contests, provide bystanders with critical information about the outcomes of others’ aggressive encounters [12]. This capacity goes beyond the previous experience of the subjects as, by using unfamiliar stimuli, we show that geladas are able to generalize the actual social value associated with vocal interactions. In particular, these primates might interpret call exchanges as meaningful third-party social interactions rather than merely as a series of independent vocal events [28].

Importantly, even though future research is needed, this field experiment unveils the proximate causes leading to prosocial behaviours after conflicts as well as that geladas might use vocal affiliation in reconciliation and consolation-like behaviours towards victims [37,38]. Altogether, this suggests a greater complexity in the functional, intentional or expressive, role of the species positive calls [29,34,45,46]. Vocalizations produced by receivers play indeed a crucial role in inducing prosocial behaviours [15] and, when produced by donors of prosocial acts, can significantly influence the mammal hormonal systems regulating social bonding, comparable to the effects of a physical contact [32,33]. Notably, here we cannot draw conclusions on the processes underlying conflict-resolution strategies as well as on how the vocal exchanges used are interpreted by listeners. Indeed, both human and non-human animal literature shows an ongoing debate on whether prosocial behaviours such as affiliation provided to victims of aggression underlie sympathetic concern or more self-protective and risk-mitigating reasons [77–79]. Moreover, although here we show that vocal exchanges in a certain sequential order attract more interest by the tested animals, future research investigating the presence of social norms [80] related to conflict-resolution strategies should investigate whether subjects are being rewarded/punished according to their perceived propensity to cooperation, as it occurs in cooperative breeding species [28].

Geladas were also affected by the emotional arousal conveyed the male positive call in the vocal exchange. Indeed, the study subjects seemed to remain more vigilant (i.e., stopped grazing behaviours, see Methods) and showed more interest to the loudspeaker when the sequence broadcast contained moans, conveying higher emotional arousal compared to grunts. Vocalisations are known to be optimal candidates for emotion transmission [81], as they show a wide variability of between- and within-call-type features allowing to transmit a graded information about one’s affective state [3–5]. The emotional nature (i.e., valence and arousal [82]) of different call variants can be adaptively (consciously or not) discriminated by a recipient, who can differently react to the stimuli with variable “emotional content” [2]. Our experiment indicates that male geladas also recognized the emotional arousal encoded by their different affiliative calls, in line with evidence suggesting gelada females’ preference towards sequence containing a derived affiliative call [46]. Emotion recognition has been considered either a prerequisite [2] or an indicator [83] for the occurrence of emotional contagion based on the perception of vocalisations (for a review on issues in the study of empathy-related phenomena, see [77]). Changes in the behavioural response of animals should indeed also be accompanied by changes in indicators of internal affective states [84]. In our study, self-directed behaviours remained consistent across experimental conditions, effectively ruling out the possibility that variations in anxiety levels [59,85] influenced by different stimuli could confound the subjects’ recognition abilities. Nevertheless, our result also suggests emotion and prosocial behaviour recognition do not systematically lead to emotional contagion [2,77,83], possibly implying that brain processes leading to mental representation can be at play [75].

In this study, we demonstrate that wild monkeys exhibit different responses to vocal interactions depending on the sequential order of calls, as well as their sensitivity to the positive call used in potential post-conflict interactions. It is important to note that our results do not allow us to distinguish between cognitive and emotional processes that may underlie their reactions. For example, we cannot conclusively determine if their responses are driven by higher-level cognitive processes, such as reference-making, or by more basic emotional responses, such as emotional recognition. Additionally, as is the case in most animal cognition experiments conducted in natural environments, our study is limited by a small sample size. The wild setting in which this study took place provides a more ecologically valid environment but also introduces variability due to individual differences, environmental conditions, and other factors that could influence the results. Additionally, the stimuli used in this experiment were derived from a captive population, and we may expect subtle inter-population differences in vocal production that could affect the generalizability of our findings.

In conclusion, this field experiment contributes to the growing body of evidence that animals use vocal cues from third-party interactions as valuable sources of information. Additionally, it underscores that the selective pressures of complex social environments may have favoured the development of cognitive abilities that represent evolutionary precursors to certain aspects of human cognition. Our findings underscore the imperative ability of animals to quickly recognize the positive *vs* negative nature of communicative exchanges between conspecifics for their survival, aiming to better understand how perception systems organize sensory information for rapid recognition. It opens new scenarios for future research employing playback experiments in naturalistic conditions to investigate mental processing and social cognition in non-human animals.

## Supporting information

S1 Fig The study site in Debre Libanos, Ethiopia. Pictures of a) the area where playback experiments were carried out (Set Deber, Debre Libanos, Ethiopia; picture by LP) and b) the relative position of the study subject while feeding and about to receive the stimulus and the experimenter (LP) hidden with the loudspeaker behind the vegetation and in a location not visible to the tested animal (picture by EP).
(TIFF)

S1 Table Ethogram of behaviours coded in the present study. (DOCX)

S1 Dataset Full raw dataset used for the statistical analyses of the study. (XLSX)
