# Supplementary material for: Wild gelada monkeys detect emotional and prosocial cues in vocal exchanges during aggression
Source: PLoS One. 2025 May 14;20(5):e0323295. doi: 10.1371/journal.pone.0323295 (PMC12077720; doi:10.1371/journal.pone.0323295)
Supplement: S1 Table — (DOCX) [file pone.0323295.s002.docx]

| **Behaviour** | **Definition** | **Reference(s)** |
| --- | --- | --- |
| Looking towards the loudspeaker | The subject lifts its head from the feeding position and orients it in the direction of the loudspeaker. | Lemasson et al., 2008; Leroux et al., 2023 |
| Self-directed behaviours (scratching, self-grooming) | Repeated movement of the hand or foot during which the fingertips are drawn across the individual’s fur (i.e., scratching) or instances when an individual grooms itself continuously (i.e., self-grooming). | Duboscq et al., 2016; Palagi et al., 2018 |
| Feeding interruption | The subject, during feeding, lifts its head and pauses the typical hand movements associated with gelada grazing behaviour for more than 0.5 seconds. In all cases, head raising was accompanied by a halt in hand movements. During these interruptions, subjects may either direct their gaze toward the loudspeaker or not. | Allan & Hill, 2018; Dunbar, 1977; Dunbar & Bose, 1991; Truppa et al., 2024 |
